# Modulation of Osteogenesis by Conditioned Media of Differently Activated Macrophages: A Comparative In Vitro Study

**DOI:** 10.3390/ijms27209185

**Published:** 2026-10-16

**Authors:** Stevo J. Najman, Jelena Živković, Sanja Stojanović

**Affiliations:** 1 Faculty of Medicine, Department of Biology with Human Genetics, University of Niš, 18108 Niš, Serbia; jelena.zivkovic.biologija@medfak.ni.ac.rs (J.Ž.); sanja.stojanovic@medfak.ni.ac.rs (S.S.); 2 Faculty of Medicine, Scientific Research Center for Biomedicine, Department for Cell and Tissue Engineering, University of Niš, 18108 Niš, Serbia

**Keywords:** macrophage activation, conditioned media, RAW 264.7 macrophages, MC3T3-E1 preosteoblasts, osteogenic differentiation

## Abstract

Macrophages play an important role in bone regeneration by releasing bioactive factors that regulate the behavior of osteoprogenitor cells. In this in vitro study, we investigated how conditioned media (CM) derived from RAW 264.7 macrophages treated with lipopolysaccharide (LPS; 1 or 100 ng/mL), dexamethasone (DEX; 10^−6^ M), or left untreated, modulate the proliferation and osteogenic differentiation of MC3T3-E1 preosteoblasts. Exposure to LPS and DEX produced different patterns of macrophage activation, assessed by microscopic morphology, nitroblue tetrazolium (NBT) reduction, and measurement of TNF-α secretion from macrophages. In the L929 bioassay, no CM was cytotoxic, while effects on MC3T3-E1 proliferation and differentiation varied with macrophage treatment and CM dilution. CM obtained from DEX-treated macrophages elicited the most pronounced pattern of osteogenic activity that included reduced cell proliferation, acquisition of osteoblast-like morphology, enhanced *von Kossa* staining, and pronounced osteocalcin and osteopontin immunoexpression. CM from untreated macrophages also supported osteogenesis, although to a lesser extent. At 100%, CMs from LPS-treated macrophages supported greater preosteoblast proliferation than other CMs, while their effects on differentiation depended on LPS dose and CM dilution. These findings indicate that macrophage-derived signals differentially modulate preosteoblast proliferation and differentiation depending on their functional state. This study provides a valuable in vitro cell model for the development of immunomodulatory and regenerative strategies in bone tissue engineering and broader applications in wound healing.

## 1. Introduction

Bone regeneration and repair are tightly regulated processes that require coordinated crosstalk between immune and skeletal cells. Among immune cells, macrophages are key modulators of osteogenesis owing to their ability to adopt distinct functional states in response to their microenvironment. Macrophages with predominantly pro-inflammatory or anti-inflammatory and reparative phenotype, often described within the framework of the M1/M2 classification, have been shown to differentially influence tissue remodeling and regeneration processes, including osteogenesis. However, these categories represent simplified descriptions, since macrophage activation in biological systems represents a continuum of heterogeneous functional states rather than two discrete phenotypes [1,2].

Tissue repair progresses through partially overlapping inflammatory, reparative, and remodeling phases [1,3]. During the inflammatory phase, cytokines and growth factors recruit immune and progenitor cells to the site of tissue damage. Macrophages are considered to be major cellular mediators of this phase, producing pro-inflammatory cytokines, growth factors, and reactive oxygen species (ROS) that shape the early healing environment and contribute to debris clearance and the recruitment of mesenchymal progenitors, whereas sustained or excessive inflammation may impair healing [1,2,4,5]. As healing progresses, macrophages can shift towards heterogeneous activation states with reparative functions and secrete factors that regulate the proliferation and differentiation of various cell types involved in tissue repair and regeneration and support angiogenesis and extracellular matrix (ECM) remodeling [1,2,3,5].

Current evidence emphasizes the role of the local immune environment, particularly macrophage-derived signals, in regulating osteoprogenitor cell behavior [2,6,7]. Pro-inflammatory macrophages are often associated with enhanced proliferation and initial recruitment of mesenchymal stem cells [6,8], and have also been reported, under certain conditions, to enhance their osteogenic differentiation and matrix mineralization [9]. Anti-inflammatory and reparative macrophages have been primarily linked to osteogenic differentiation and ECM mineralization [2,6,7]. Therefore, the temporal sequence and functional balance of macrophage activation states are considered important for successful bone healing. However, the specific effects of distinct macrophage activation states on osteogenesis remain incompletely understood [10,11,12]. This uncertainty is compounded by macrophage plasticity, as individual and combined stimuli can induce heterogeneous functional states characterized by partially overlapping, non-identical transcriptional and secretory profiles [13,14]. Although it is known that signals originating from macrophages influence osteogenic cells, comparing findings obtained using different macrophage stimuli and varying concentrations of conditioned medium (CM) across different experimental systems remains a challenge. A key contribution of the present study lies in the direct comparison of early responses in MC3T3-E1 cells to CM derived from RAW 264.7 macrophages treated with two concentrations of lipopolysaccharide (LPS; 1 or 100 ng/mL) or dexamethasone (DEX; 10^−6^ M), or left untreated.

Modulation of macrophage activity during the bone-healing process can alter the cytokine milieu at the injury site and may represent a strategy for improving bone regeneration outcomes, particularly in impaired or delayed healing. In vitro cell models can provide simple experimental systems for examining the effects of secreted factors from one cell type on another under controlled conditions. In that context, transfer of conditioned media (CM) from macrophage cultures to target cells provides a CM-based indirect co-culture model for examining the effects of the complete conditioned medium, without direct cell–cell contact or reciprocal signaling. The specific contribution of this study is a comparison, within the same RAW 264.7–MC3T3-E1 system, of CM derived from macrophages exposed to LPS at two concentrations or to DEX, or left untreated, and applied at several concentrations. This design addresses how both macrophage conditioning and CM dilution relate to early preosteoblast responses, without establishing the causal contribution of individual mediators.

This in vitro study aimed to investigate how CM derived from RAW 264.7 macrophages treated with two different LPS concentrations (pro-inflammatory activation conditions) or DEX (reparative-like activation condition), or left untreated, affect the proliferation and osteogenic differentiation of MC3T3-E1 preosteoblastic cells in an indirect co-culture system. The LPS-treated groups are designated as M(LPS1) and M(LPS100), and the DEX-treated group as M(GC), in accordance with the nomenclature based on the type of stimulus [15]. These designations describe the applied activation conditions rather than fully characterized, discrete M1/M2 phenotypes.

## 2. Results

### 2.1. Morphological and Functional Responses of RAW 264.7 Macrophages to Different Treatments

RAW 264.7 macrophage morphology, conditioned-medium cytotoxicity, and cellular oxidative activity were evaluated following the treatment with lipopolysaccharide (LPS) or dexamethasone (DEX).

#### 2.1.1. Morphology of Differently Activated RAW 264.7 Macrophages

Phase-contrast microscopy revealed treatment-dependent differences in the morphology and spatial organization of RAW 264.7 macrophages. In RAW 264.7 cultures treated with 100 ng/mL LPS, cells with a spread or stellate morphology were frequently observed, together with occasional elongated, spindle-shaped and fibroblast-like cells. Some cells contained phase-bright intracellular vacuole-like structures. Rare cells appeared to be binucleated. Cultures treated with 1 ng/mL LPS appeared more morphologically heterogeneous and comprised round, polygonal, stellate, elongated and fibroblast-like cells, as well as occasional large, flattened cells with vesicles and vacuoles. Stellate and spread cell morphologies were less frequent in DEX-treated cultures and were least apparent in untreated control cultures, in which round or polygonal cells predominated, and only rarely contained vesicles or vacuoles. Cell aggregates of various shapes and numbers were observed in all groups. At lower magnification, cells appeared more dispersed in cultures treated with 100 ng/mL LPS, whereas clustering was more evident in the 1 ng/mL LPS group. DEX-treated cultures contained particularly dense and compact aggregates, while the untreated control cultures displayed a comparatively more uniform distribution of densely packed cells (Figure 1a).

#### 2.1.2. TNF-α Secretion by Differently Activated RAW 264.7 Macrophages

TNF-α concentrations differed significantly among the experimental groups. Compared with Untreated-CM, TNF-α concentrations were significantly increased in both the M(LPS100) and M(LPS1) groups and significantly decreased in the M(GC) group (*p* < 0.001 for all comparisons). TNF-α concentration was also significantly higher in M(LPS100) than in M(LPS1) group (*p* < 0.001) (Figure 1b).

#### 2.1.3. Treatment-Dependent NBT Reduction in RAW 264.7 Macrophages

Formazan production, resulting from NBT reduction in NBT assay, correlates with intracellular ROS production (specifically the superoxide anion) in macrophages and provides a semi-quantitative assessment of their oxidative activity. Formazan production was detected under all experimental conditions, with the highest mean production observed in macrophages treated with 100 ng/mL of LPS, followed by those treated with 1 ng/mL of LPS, untreated macrophages, and DEX-treated macrophages (Figure 1c).

#### 2.1.4. Effects of Macrophage-Derived CM on L929 Cell Viability and Morphology

According to the results of the MTT test, the viability of L929 cells cultured in all CM remained comparable to or higher than that of the DMEM control, indicating that none of the CM exhibited a cytotoxic effect on L929 cells (Figure 2a). The lowest mean MTT reduction was observed in cells cultured in 50% M(LPS100)-CM. However, although CM concentration affected viability, this effect did not show a uniform pattern when looking at different groups. In the M(LPS100)-CM group, the lowest mean viability was observed with 50% CM, and significant differences between CM concentrations were observed in both M(LPS1)-CM and M(GC)-CM. Microscopic analysis revealed high cell density and near-confluent monolayers in all examined groups (Figure 2b). Cultures grown in CM from macrophages treated with 1 ng/mL LPS appeared slightly less dense than the other CM-treated cultures. Cells grown in CM derived from LPS-treated macrophages exhibited a predominantly elongated, spindle-shaped morphology, with some cells displaying thin cytoplasmic extensions. Microscopic examination of the different CM concentrations did not reveal a uniform dilution-dependent change in L929 cell morphology or density. The 50% M(LPS100)-CM culture appeared slightly less dense than the corresponding 100% and 25% conditions, consistent with the lower mean viability obtained by MTT assay at this concentration.

### 2.2. Osteogenic Differentiation of MC3T3-E1 Cells in Conditioned Media

#### 2.2.1. Effect of Macrophage-Derived Conditioned Media on MC3T3-E1 Relative Cell Number

The results of the MC3T3-E1 crystal-violet (CV) assay, an indirect estimate of adherent-cell biomass or relative cell number, are presented in Figure 3. At 100% CM, all CM groups showed lower average proliferation compared to DMEM control. An exploratory two-factor repeated-measures ANOVA of four repetitions, with macrophage condition and CM concentration as factors, yielded a nominally significant effect of macrophage condition (F(3, 9) = 16.46, *p* = 0.0164), a nominally significant effect of CM concentration (F(2, 6) = 47.65, *p* = 0.00155), and a nominally significant interaction between the two factors (F(6, 18) = 12.53, *p* = 0.0196; Greenhouse–Geisser-corrected *p* values). At 25% CM, M(LPS100)-CM showed a lower relative CV signal than M(LPS1)-CM (Holm-adjusted *p* = 0.0018) and M(GC)-CM (*p* = 0.0087). Its comparison with Untreated-CM was not significant after Holm correction (*p* = 0.0608). The significance symbols in Figure 3 refer to the previously applied one-way ANOVA and its post hoc comparisons, whereas the F statistics and Holm-adjusted comparisons reported here are from the two-factor repeated-measures ANOVA. These *p* values describe variation among repetitions in the analyzed series.

CM dilution modified MC3T3-E1 proliferation differently, depending on the state of macrophages, i.e., the CM group tested. In the M(LPS100)-CM group, 25% CM led to a significant decrease in proliferation compared to DMEM control, and also showed significantly lower proliferation compared to 100% and 50% CM, as well as compared to the same concentrations of M(LPS1)-CM and Untreated-CM. On the other hand, in the M(LPS1)-CM and M(GC)-CM groups, 50% and 25% CM showed significantly higher proliferation compared to the corresponding 100% CM. In the Untreated-CM group, 50% CM showed the highest average proliferation. Among the concentrations of 100% CM, M(GC)-CM showed significantly the highest proliferation inhibition compared to all other CM groups.

#### 2.2.2. Microscopic Analysis of MC3T3-E1 Cells Cultured in Conditioned Media on Days 3 and 6

For the microscopic assessment of osteogenic differentiation, 100% and 25% CM concentrations were examined. After 3 days of culture (Figure 4a), MC3T3-E1 cells maintained in all conditioned media (CM), as well as in osteogenic medium (OM), exhibited a predominantly prismatic morphology with filopodial extensions, forming intercellular connections through cellular protrusions. In cultures incubated with 100% M(GC)-CM, cells frequently formed circular arrangements, which were more prominent than those observed in the OM group. The cell density in this group was also notably higher compared with both OM and DMEM groups. Cells treated with other CM exhibited morphological characteristics similar to those observed in OM group. However, cultures treated with Untreated-CM appeared to have a slightly higher cell density. Compared with the corresponding 25% M(GC)-CM, the 100% CM culture also appeared more densely populated and showed more prominent spatial organization.

After 6 days of culture (Figure 4b), pronounced changes in cell morphology and spatial organization were observed compared with those observed on day 3. Cell cultures maintained in all CMs exhibited a high cell density and were confluent or nearly confluent. Cultures cultivated in M(GC)-CM appeared most confluent (especially at 100% concentration), followed by those cultivated in Untreated-CM. In the M(GC)-CM group, the predominant cell population comprised large, polygonal cells characterized by prominent nuclei and lamellipodial extensions. In contrast, cultures grown in other CMs exhibited a heterogeneous cell population, with cells displaying both lamellipodial and filopodial extensions. Although differences between the 100% and 25% CM were apparent in some groups, particularly M(GC)-CM, these concentration-related differences in cell density appeared less pronounced by day 6 than on day 3.

#### 2.2.3. Assessment of Osteogenic Differentiation of MC3T3-E1 Cells Cultured in Conditioned Media

*Von Kossa* staining, used as an indicator of calcium phosphate (CaP) deposition, was positive in all examined groups. Compared with cells maintained in DMEM, the extent of CaP deposition, as indicated by *von Kossa* staining, appeared greater in all CM groups, as well as in the OM group. The most intense staining was observed in the cultures cultivated in M(GC)-CM and M(LPS100)-CM, followed by Untreated-CM. In contrast, cultures grown in M(LPS1)-CM exhibited slightly weaker staining than the OM group (Figure 5). Comparison of the two CM concentrations showed that *von Kossa*-positive deposits remained clearly detectable at 25% CM. Although differences between 100% and 25% CM were apparent within individual groups, dilution did not produce a uniform reduction in staining across the different CM groups.

Osteopontin (OPN) immunoreactivity was detected in MC3T3-E1 cells in all CM and control groups but at different extent. Qualitative and quantitative examination indicated that OPN immunostaining appeared most pronounced in cultures grown in CM derived from untreated macrophages, followed by cultures grown in M(GC)-CM and M(LPS100)-CM. Comparatively weaker immunopositivity was observed in cultures grown in M(LPS1)-CM and in the control groups (Figure 6). OPN immunoreactivity remained detectable at both 100% and 25% CM concentrations. Differences in apparent staining intensity between the two concentrations varied among CM groups, with no consistent concentration-dependent pattern.

Osteocalcin (OCN) immunoreactivity was detected in all examined groups but at different extent. Qualitative examination indicated that OCN immunostaining appeared most pronounced in cultures grown in M(GC)-CM, as confirmed also by ICC OD score. Weaker immunoreactivity was observed in cultures grown in Untreated-CM and M(LPS100)-CM. Cultures grown in M(LPS1)-CM, as well as control cultures grown in OM only, showed relatively weak OCN staining (Figure 7).

Comparison of the 100% and 25% CM conditions did not reveal a uniform concentration-dependent pattern across all CM types for *von Kossa*, OPN, or OCN staining. Notably, OCN immunoreactivity remained pronounced in 25% M(GC)-CM condition, indicating that osteogenic-associated staining remained clearly evident after dilution in this group.

## 3. Discussion

### 3.1. LPS and DEX Effects on RAW 264.7 Macrophages In Vitro

In this in vitro study, RAW 264.7 macrophages were treated with LPS or DEX to establish pro-inflammatory M(LPS) and DEX-conditioned, reparative-like M(GC) conditions, respectively, for subsequent evaluation of the effects of their conditioned media on the osteogenic differentiation of MC3T3-E1 preosteoblastic cells. These stimulus-based terms describe the applied experimental conditions and associated macrophage responses rather than definitive polarized phenotypes, as macrophage activation is a continuous stimulus- and time-dependent process [15]. The results of our preliminary experiments on resident peritoneal macrophages indicated a dose-dependent effect of LPS on macrophage pro-inflammatory activation. Therefore, RAW 264.7 macrophages were treated with the highest and lowest LPS concentrations tested (100 ng/mL and 1 ng/mL). Dexamethasone (DEX) was used with the aim of inducing a reparative-like M(GC) condition [16,17]. A reparative phenotype was not confirmed within the framework of the presented protocol. Treatments were conducted for 4 h, a duration shown to be sufficient to initiate macrophage activation responses [18]. Following treatment, the media containing reagents used for activation were replaced with fresh DMEM, and cells were incubated for an additional 20 h for subsequent collection of factors secreted from activated macrophages in CM. CM were then collected and later used for in vitro osteogenic differentiation experiments and other investigations. This approach minimized the direct effects of LPS and DEX on osteogenic differentiation, so that the observed outcomes were intended primarily to reflect the influence of macrophage-derived secretory factors.

Microscopic analysis revealed that macrophages treated with 100 ng/mL LPS exhibited morphological features consistent with pro-inflammatory activation conditions, including a stellate shape with numerous extensions, cytoplasmic vesicles/vacuoles, and the appearance of binucleation [19,20,21]. These morphological changes were accompanied by the highest mean formazan production in the 100 ng/mL LPS-treated macrophages. Formazan-based NBT tests have been used as indirect indicators of cellular oxidative activity [22], although it should be noted that NBT reduction predominantly reflects intracellular superoxide formation and may depend on cell number, incubation time, and stimulus intensity [23]. Formazan levels in the macrophages exposed to 1 ng/mL LPS were lower than in the 100 ng/mL LPS group, but higher than in untreated or DEX-treated cells. This provides evidence supporting the concentration-dependent nature of macrophage activation by LPS [24,25].

It is well-established that LPS stimulates macrophages to produce TNF-α, a key pro-inflammatory cytokine [20,26,27]. In our ELISA measurement, the highest mean TNF-α concentration was detected in CM from macrophages exposed to 100 ng/mL LPS, followed by 1 ng/mL LPS, both of which were statistically higher than the mean concentration in CM from untreated macrophages, but there was also a statistically significant difference between the two M(LPS)-CMs. This finding is consistent with Borzęcka et al. [28], who reported increased TNF-α secretion following stimulation of RAW 264.7 macrophages with LPS concentrations ranging from 1 to 1000 ng/mL. Also, increased TNF-α production following LPS stimulation has been consistently observed in the RAW 264.7 model, as demonstrated by the systematic review and meta-analysis conducted by Facchin et al. [29]. These findings support the use of TNF-α as a reliable marker of LPS-induced inflammatory activation in RAW 264.7 macrophages.

On the other hand, M(GC)-CM had significantly lower TNF-α concentrations compared to untreated macrophages. These differences support the intended pro-inflammatory character of the M(LPS) conditions and the reduced pro-inflammatory character of the M(GC) condition.

M(GC) macrophages exhibited a morphology similar to that of untreated cells, except that they were slightly larger, predominantly round, and occasionally had short extensions, which are features compatible with the applied reparative-like condition [18,21]. Cells from the control group were round in shape and smaller, which is characteristic of untreated macrophages [18,21,30,31,32]. The formazan production observed in the untreated group likely reflects basal ROS production by RAW 264.7 macrophages [33]. Unstimulated RAW 264.7 macrophages should not be considered functionally inactive, as they can exhibit basal inflammatory activity. Stojanović and Najman [34] demonstrated that inflammatory mediator expression and secretion in unstimulated RAW 264.7 macrophages could be further modulated by stem-cell-derived conditioned media. This basal activity is consistent with the detectable TNF-α concentration and NBT reduction observed in our Untreated group. However, macrophages cultured in DMEM produced higher levels of formazan compared to those treated with DEX, consistent with the fact that reparative M(GC) conditions are accompanied by reduced oxidative activity [27]. Published findings indicate that TNF-α production is reduced in macrophages treated with DEX [35], which is consistent with the lower mean TNF-α concentration measured in our M(GC)-CM. None of the CMs tested, including M(LPS)-CMs with higher measured TNF-α concentrations, exhibited a cytotoxic effect on L929 cells under the conditions of our experiment. Although some differences in L929 cell viability were observed among CM concentrations, the elevated TNF-α levels measured in M(LPS)-CMs were not associated with detectable cytotoxicity.

The absence of cytotoxicity following exposure to macrophage-CM was compatible with higher TNF-α levels in M(LPS)-CM and higher NBT reduction in LPS-treated macrophages. LPS stimulation induces a pro-inflammatory response in RAW macrophages, increasing TNF-α secretion and oxidative activity, particularly at higher LPS concentrations [28,36]. However, TNF-α is not intrinsically cytotoxic, as its biological effect depends on its concentration, exposure duration, the susceptibility of the target cells, and the presence of additional death-promoting signals. At sublethal concentrations, TNF-α primarily activates inflammatory and immunomodulatory signaling rather than cell-death mechanisms [37]. Thus, elevated NBT reduction in LPS-treated macrophages was not accompanied by L929 cytotoxicity of their CM under the conditions tested.

Overall, RAW 264.7 macrophages cultured with LPS, DEX, or maintained without treatment, exhibited distinct morphological, oxidative, and secretory response patterns. Within the scope of this in vitro study, these differently treated and untreated macrophages served as sources of CM for determining whether their secreted factors—present in the CM—differentially modulate the osteogenic differentiation of MC3T3-E1 preosteoblastic cells.

### 3.2. Modulatory Effect of Macrophages on Osteogenic Cell Differentiation In Vitro

Since cytotoxicity assays demonstrated that the conditioned media (CM) did not adversely affect cell viability, according to our study design, the CMs were subsequently evaluated for their effects on the osteogenic differentiation of a preosteoblastic cell line [38]. Cell proliferation as adherent-cell biomass was assessed in parallel because proliferation and differentiation are temporally and functionally coordinated during the process of osteoblastogenesis [39,40].

The CV-based proliferation assay is a simple assay used to obtain quantitative information on the relative density of adherent cells or adherent-cell biomass, because the amount bound dye is proportional to cell number and therefore this assay was used here as an indirect assessment of cell proliferation [41,42]. All tested CMs at 100% concentration resulted in lower proliferation of MC3T3-E1 preosteoblast cells compared to control cells cultured in DMEM alone. The interaction in the exploratory two-factor analysis suggests that dilution altered the relative CV signal differently across macrophage conditions. However, dilution of CM modified the proliferative response specifically for the CM group, i.e., there was no uniform concentration-dependent effect. A 25% dilution of M(LPS100)-CM led to a significant decrease in proliferation, while dilutions of M(LPS1)-CM and M(GC)-CM of 50% and 25% were associated with higher proliferation than the corresponding 100% CM. Among the 100% CM groups, the estimated proliferation rate was higher in cells cultured in M(LPS)-CMs than in those cultured in M(GC)-CM or Untreated-CM. The lowest mean proliferation rate among the 100% CM groups was observed in the M(GC)-CM group, which showed the greatest reduction in cell proliferation; this reduction was statistically significant compared with both the DMEM control and M(LPS)-CMs. It is notable that M(LPS)-CMs, which supported greater cell proliferation than the other CMs tested, also had a higher mean concentration of TNF-α, and that M(GC)-CM had significantly lowest TNF-α concentration among all tested CMs. Because TNF-α was measured in the original undiluted CM, its effective concentration in the 50% and 25% CM conditions would be expected to decrease proportionally, together with other soluble CM components. Therefore, the dilution-dependent CV signal in MC3T3-E1 cells may reflect changes in the complete medium, including secreted factors, nutrients, and metabolites, rather than a specific action of TNF-α. At physiological concentrations, TNF-α may stimulate cell growth and proliferation, as previously demonstrated in studies conducted on murine intestinal cells [43]. Furthermore, Bousch et al. [44] showed that proinflammatory cytokines, including IL-1β and TNF-α, promoted the proliferation of human osteoblast-like cells. Similarly, He et al. [45] investigated the effects of CM derived from proinflammatory, anti-inflammatory, and resting/unpolarized macrophages on bone marrow mesenchymal stem cells (BMSCs). Their findings indicated that CM from proinflammatory macrophages had the most beneficial effect on BMSCs proliferation compared with CM from resting/unpolarized macrophages and, particularly, anti-inflammatory macrophages. Taken together, the association observed in our study is consistent with previous reports suggesting that TNF-α and other macrophage-derived proinflammatory signals may support osteoprogenitor proliferation [44,45]. However, because CM contains multiple bioactive factors representing a complex mixture of cytokines, growth factors, and metabolites, and their neutralization procedures, nor blockades of their receptors were not performed in the present study, the observed cellular responses cannot be causally attributed solely to TNF-α, but rather reflect the collective biological activity of the whole secretome.

Testing multiple concentrations of CM proved particularly informative in this study, as the interaction in the exploratory two-factor analysis suggested that dilution modified the MC3T3-E1 CV signal in a manner that depended on the activation state of the macrophages. The significantly more pronounced reduction in proliferation observed with 25% M(LPS100)-CM was in contrast to the more pronounced proliferation observed after the same dilution of M(LPS1)-CM and M(GC)-CM compared to their respective 100% concentrations. This is consistent with Liang et al. [46], who showed that BMSC proliferation and osteogenic differentiation were influenced by both macrophage phenotype and CM concentration, and with Cai et al. [47], who evaluated different concentrations of macrophage-CM before selecting 50% CM for subsequent osteogenic experiments. Therefore, CM concentration should be considered a biologically relevant experimental variable and not just a simple technical parameter. Because CM was diluted with DMEM, nutrient and metabolite concentrations also changed and the responses cannot be assigned solely to macrophage-derived factors or TNF-α. In the subsequent phase of the experiments, we investigated the effect of CMs, supplemented with osteogenic factors, on the osteogenic differentiation of MC3T3-E1 cells. In these differentiation experiments, 100% and 25% CMs were examined. Qualitatively, the effect of CM concentration was not uniform across different macrophage conditions. Cells cultured in OM or DMEM were used as controls. In order to characterize the osteogenic phenotype, upon reaching confluence after 6 days of cultivation, CaP clusters were stained by the *von Kossa* method, while the production of OPN and OCN was determined by ICC staining. It is known from the literature that the synthesis of OPN is increased during the phase of active osteoblast proliferation, and in the postproliferative phase it decreases, but increases again during the phase of ECM mineralization. In contrast, osteoblasts express OCN only in the postproliferative phase, and the maximum expression of this protein is observed during the mineralization phase, so it is considered to be a marker of mature osteoblasts [48].

Microscopic findings further supported the results of testing different concentrations of CM. Dilution did not uniformly attenuate the biological effects of CM. In L929 cultures, cell morphology and high cell density were generally preserved at all concentrations tested, consistent with the absence of apparent cytotoxicity. In MC3T3-E1 cultures, concentration-related differences in cell density and organization were more apparent at day 3, especially for M(GC)-CM, while these differences became less pronounced by day 6 as the cultures approached confluence. Moreover, *von Kossa*, OPN, and OCN staining remained detectable at 25% CM, and dilution did not result in a consistent reduction in staining associated with osteogenic processes across all CM groups. Similar concentration-dependent effects of macrophage-derived CM on osteoprogenitor proliferation and osteogenic differentiation have been previously reported [46,47]. These observations suggest that decreasing the concentration of CM may modify certain cellular responses without proportionally reducing all biological effects of the macrophage secretome.

Immunocytochemical (ICC) analysis of MC3T3-E1 preosteoblasts cultured in the presence of different CMs revealed OPN expression in all experimental and control groups. MC3T3-E1 cells are capable of undergoing a time-dependent sequence of osteoblastic differentiation and extracellular matrix formation [40]. The detection of OPN, a non-collagenous ECM protein associated with osteoblastic differentiation, in all cultures is therefore consistent with the osteoblastic phenotype of this cell line [49].

Preosteoblasts cultured in M(LPS100)-CM exhibited higher apparent cell density and more pronounced *von Kossa* staining than the OM and DMEM controls, while OPN and OCN immunoreactivity, was comparable or slightly weaker to that observed in control groups. In contrast, M(LPS1)-CM was associated with the weakest OPN and OCN immunoreactivity, together with comparatively limited *von Kossa* staining among CM-treated groups. The differences between the two M(LPS)-CM groups can be considered in relation to their measured TNF-α concentrations: the M(LPS100)-CM had the highest mean concentration, whereas the M(LPS1)-CM had a more moderate, but statistically higher mean concentration than Untreated-CM. Because osteogenic differentiation was assessed using both 100% and 25% CMs, dilution reduces the effective concentrations of TNF-α as well as other soluble components of the secretome, supporting the interpretation that the observed responses reflect the complete composition of the CM and not just TNF-α. Earlier in vitro studies demonstrated that TNF-α suppresses alkaline phosphatase activity, OCN and type I collagen expression, and impairs ECM mineralization [48]. Conversely, recent findings [44] suggest that pro-inflammatory cytokines, including TNF-α, can promote osteogenesis. These apparently divergent effects may depend on cytokine concentration, exposure duration, receptor signaling, other CM components, and the cellular model. Therefore, the present results show an association between CM TNF-α concentration and the MC3T3-E1 response, but do not demonstrate that TNF-α alone caused the observed osteogenic patterns.

Notably, M(GC)-CM was associated with the most pronounced osteogenic response. Preosteoblasts cultured in this CM formed distinctive circular arrangements by day 3 and exhibited osteoblast-like morphology by day 6 of osteogenic differentiation, compared to all other experimental and control groups. These cultures also demonstrated more pronounced *von Kossa* staining and OCN immunoreactivity. It is important to note that OCN ICC OD score revealed that osteogenic characteristics were observed with M(GC)-CM at both concentrations tested, indicating that its effect is not limited to undiluted CM. While M(GC)-CM did not exert cytotoxic effects on L929 cells, it produced the lowest mean proliferation value among the 100% CMs. This observation aligns with literature reports which indicate that osteoblast maturation is associated with a decline in proliferative capacity, but increased synthesis of ECM components [50]. These findings indicate that the M(GC) condition was associated with the most pronounced osteogenic pattern under the tested conditions.

Similarly, preosteoblasts cultured in Untreated-CM exhibited osteoblast-like morphology by day 6 of osteogenic differentiation. These cultures showed a modest increase in *von Kossa* staining, and OCN immunoreactivity, as well as more evident increase in OPN reactivity than OM and M(LPS1)-CM group, although the overall osteogenic pattern was less pronounced than with M(GC)-CM. Additionally, CM from untreated macrophages was associated with a lower mean proliferation value of MC3T3-E1 cells, which, in conjunction with the observed differentiation markers, suggests an overall promotion of osteogenic differentiation. These findings are consistent with our previously published in vivo study, in which untreated peritoneal macrophages were used as components of ectopic implants to observe the osteogenic process, and which demonstrated the highest degree of osteogenesis compared to other types of implants without macrophages [51].

Likewise, other authors showed that mesenchymal stem cells (MSCs) co-cultured with undifferentiated or anti-inflammatory macrophages had significantly higher ALP activity than those co-cultured with pro-inflammatory macrophages. Nevertheless, all macrophage-MSC co-cultures showed enhanced bone ECM mineralization, in comparison with MSCs cultured alone. The most pronounced mineralization was observed in the pro-inflammatory macrophage-MSCs co-culture. Despite an initial inhibition of osteogenesis, this co-culture type exhibited the highest degree of bone formation in four weeks. Given that MSCs support the anti-inflammatory macrophage phenotype, it is possible that a phenotypic transition from pro-inflammatory to anti-inflammatory macrophages occurred over time [9]. Another study [52] demonstrated that CM from macrophages induced toward anti-inflammatory macrophage polarization via bone morphogenetic protein 2 (BMP2) and CaP cement (CPC) combination enhanced MSC osteogenic differentiation, while in vivo, BMP2-CPC facilitated bone regeneration by increasing local anti-inflammatory macrophage presence and reducing inflammation.

These observations from the literature, along with our results, highlight the importance of an initially pro-inflammatory macrophage response in the early phase of osteogenesis, followed by a timely transition to an anti-inflammatory phenotype in order to fully support the osteogenic process.

### 3.3. Clinical Implications and Broader Relevance to Wound Healing

The present results have potential translational relevance because they suggest that macrophage-derived signals can differentially support osteoprogenitor proliferation and osteogenic maturation. For bone defects and complex wounds, the results suggest that different macrophage signals may be beneficial at different stages of healing. Early pro-inflammatory signals may promote cell recruitment and proliferation, whereas later reparative-like signals may support matrix formation, vascularization, and tissue maturation [3,53,54]. Accordingly, macrophage-loaded biomaterials, sustained delivery systems, or selected secretome components could be explored as a complement to tissue engineering approaches. However, the present findings do not support direct clinical use of LPS, DEX, or uncharacterized CM, as translation would require identification and standardization of the active factors, dose and timing assessment, testing with primary human cells, and validation in relevant in vivo models.

In addition to bone regeneration, the model has broader value for wound healing research, as macrophage dysfunction can disrupt overlapping inflammatory, proliferative, and remodeling phases, and chronic wounds typically retain a persistent pro-inflammatory macrophage profile and fail to effectively progress toward repair [55,56]. The in vitro system used in this study and the results obtained could be applied to studies on wound healing and chronic wounds. Using different activation agents and concentrations to establish differently stimulated macrophage conditions may help develop wound-healing models and evaluate candidate interventions. The macrophage-CM/target-cell platform provides a reductionist screening system in which stimulus, concentration, exposure time, and the response of a defined target cell can be examined separately before moving to co-culture or animal models. With appropriate adaptation, it could be applied to fibroblasts, endothelial cells, keratinocytes, or patient-derived progenitor cells to test how macrophage secretomes affect proliferation, migration, angiogenesis, extracellular matrix remodeling, and lineage differentiation. Its main limitation is the absence of reciprocal cell–cell signaling and the complete wound microenvironment and therefore, the promising CM effects should be subsequently examined in multicellular models both in vitro and in vivo.

Taking together, our findings support the differential effects of M(LPS)-CM and M(GC)-CM on osteogenesis, but this single-stage CM experiment, due to several limitations, does not demonstrate the temporal transition from a proinflammatory to a reparative macrophage phenotype. The Day 6 time point was selected to assess early responses associated with osteogenesis, at a stage when the MC3T3-E1 cell cultures treated with CM were confluent or nearly confluent. It does not indicate terminal osteoblast maturation or ongoing matrix mineralization. Future studies should include additional macrophage phenotypic markers and later time points of the osteogenic process.

### 3.4. Study Limitations

A significant limitation of this study is its reliance on a simplistic, reductionist in vitro model of indirect coculture, based on the mouse cell lines RAW 264.7 and MC3T3-E1. Although this system allows a controlled assessment of the effects of conditioned medium (CM) on the osteogenic response, the use of murine cell lines limits the ability to extrapolate the results to primary human cells. The model of CM transfer does not include direct contact between cells, reciprocal signaling and continuous exchange of soluble factors, as well as other conditions which are characteristic of the bone healing process in vivo. Consequently, the observed in vitro responses cannot be directly extrapolated to in vivo bone regeneration. The Day 6 endpoint does not capture late osteoblast maturation or long-term mineralization. Staining analyses based on images are semi-quantitative, and crystal violet absorbance provides an indirect estimate of the number of adherent cells rather than a direct measure of cell proliferation or a shift toward differentiation. Macrophage conditions were defined based on the applied stimuli, and their morphology, NBT reduction, and TNF-α secretion did not provide a comprehensive phenotypic characterization without using a broader panel of markers. The effects of individual soluble mediators, including TNF-α, were not investigated via neutralization assays, nor was the viability of MC3T3-E1 cells directly assessed. Longer-term studies should include additional phenotypic and functional assays.

## 4. Materials and Methods

### 4.1. Cell Lines and Culture Conditions

Murine cell lines, including RAW 264.7 macrophages (from ATCC, Manassas, VA, USA), L929 fibroblasts (from ATCC, Manassas, VA, USA), and MC3T3-E1 pre-osteoblasts (from DSMZ, Braunschweig, Germany), were used in the experiments. All cells were cultured and maintained in Dulbecco’s Modified Eagle Medium (DMEM; PAA Laboratories, Pasching, Austria) supplemented with 10% fetal bovine serum (FBS; PAA Laboratories, Pasching, Austria), 2 mM L-glutamine, and an antibiotic-antimycotic solution (PAA Laboratories, Pasching, Austria). Cells were maintained in an incubator at 37 °C in a humidified atmosphere containing 5% CO_2_. The culture medium was replaced every 2–3 days until the cells reached the desired confluence.

### 4.2. RAW 264.7 Treatments and Conditioned Media Preparation

RAW 264.7 macrophages were seeded in 12-well culture plates (Greiner Bio-One, Frickenhausen, Germany) at a density of 2 × 10^5^ cells per well in DMEM and incubated for 24 h. After this period, the medium was removed and replaced with DMEM containing specific concentrations of lipopolysaccharide (LPS; Escherichia coli O111:B4, Sigma-Aldrich, St. Louis, MO, USA) or dexamethasone (DEX) (Galenika, Belgrade, Serbia). LPS was applied at concentrations of 100 ng/mL or 1 ng/mL [57,58,59], while DEX was used at a concentration of 10^−6^ M [60,61]. The results of our preliminary experiments on resident peritoneal macrophages indicated a dose-dependent effect of LPS on macrophage pro-inflammatory activation. Therefore, RAW 264.7 macrophages were treated with the highest and lowest LPS concentrations tested (100 ng/mL and 1 ng/mL). These concentrations were used to compare two levels of inflammatory stimulation. The concentration of 10^−6^ M was selected based on previously published suppression of inflammatory responses, including inducible nitric oxide synthase expression in RAW 264.7 cells [60,61] and a previously studied pharmacologically active concentration in in vitro experiments. In accordance with stimulus-based nomenclature, the two LPS-treated groups were designated as M(LPS100) and M(LPS1) to distinguish between the LPS concentrations, whereas DEX-conditioned macrophages were designated as M(GC) [15,16,17,62]. Macrophages were exposed to the respective stimuli for 4 h, after which the treatment media were removed and replaced with fresh DMEM, followed by an additional 20 h of incubation. Control macrophages were maintained in DMEM alone according to the same medium-change and incubation protocol (4 h + 20 h; Untreated group). Subsequently, conditioned media (CM) from M(LPS100), M(LPS1), M(GC), and Untreated groups of macrophages were collected, centrifuged at 3000 rpm for 10 min at room temperature, aliquoted, and stored in sterile vials at −20 °C until further use. The resulting conditioned media were designated M(LPS100)-CM, M(LPS1)-CM, M(GC)-CM, and Untreated-CM, respectively.

### 4.3. Nitroblue Tetrazolium (NBT) Assay

The nitroblue tetrazolium (NBT) assay was used to assess intracellular ROS production by macrophages, based on their ability to reduce NBT to blue formazan, a reaction predominantly mediated by intracellular superoxide. The assay was performed according to the method described by Choi et al. [23], with minor modifications. After CM collection, RAW 264.7 macrophages were incubated with 0.1% (*w*/*v*) NBT solution for 60 min at 37 °C. To quantify formazan formation, the intracellular crystals were solubilized in 60 µL of 2 M KOH and dimethyl sulfoxide (DMSO) (Sigma-Aldrich, St. Louis, MO, USA). Absorbance was measured at 620 nm using an automatic microplate reader (Multiskan Ascent, Thermo Labsystems, Helsinki, Finland).

### 4.4. L929 Assay for CM-Associated Cytotoxicity

The potential cytotoxicity of macrophage-conditioned media (CM) was assessed using the widely employed L929 cell cytotoxicity bioassay based on the MTT test. L929 cells were seeded in 96-well culture plates in DMEM at a density of 2.5 × 10^4^ cells per well and incubated for 24 h. After incubation, the culture medium was removed and replaced with CM or diluted CM. The 100% CM consisted of undiluted CM while 50% and 25% CM were prepared by diluting the 100% CM with DMEM at 1:1 and 1:3 ratios (*v*/*v*), respectively. Fresh DMEM was added to the control wells. Cells were then incubated for an additional 24 h. At the end of the incubation period, cytotoxicity was evaluated using MTT test.

### 4.5. MTT Test

Cellular metabolic activity, used as an indirect measure of cell viability, was assessed using the colorimetric MTT test described by Mosmann [63], with minor modifications. The assay is based on the reduction in the yellow tetrazolium salt 3-(4,5-dimethylthiazol-2-yl)-2,5-diphenyltetrazolium bromide (MTT; Carl Roth, Karlsruhe, Germany) by cellular NAD(P)H-dependent oxidoreductases in metabolically active cells, resulting in the formation of insoluble purple formazan crystals. Following incubation with conditioned media, the culture media were removed and the cells were incubated with 100 µL of MTT solution (1 mg/mL) for 3 h at 37 °C. The MTT solution was then removed, and the resulting formazan crystals were solubilized with 100 µL of 2-propanol (Thermo Fisher Scientific, Waltham, MA, USA). Absorbance was measured at 540 nm using a microplate reader. Relative cell viability, estimated from cellular metabolic activity, was expressed as a percentage of the control value and calculated using the following equation: Relative cell viability (% of control) = (mean absorbance of treated cells/mean absorbance of control cells) × 100.

### 4.6. Enzyme-Linked Immunosorbent Assay (ELISA) Determination of TNF-α

The secretion of the pro-inflammatory cytokine TNF-α by RAW 264.7 macrophages was assessed by measuring its concentration in CM collected from the M(LPS100), M(LPS1), M(GC), and Untreated groups using a Mouse TNF-α Quantikine ELISA Kit (MTA00B; R&D Systems, Minneapolis, MN, USA). The assay was performed according to the manufacturer’s instructions, and TNF-α concentrations were expressed as pg/mL.

### 4.7. Crystal Violet Assay for Indirect Estimation of Adherent Cell Number

The effects of conditioned media (CM) on MC3T3-E1 cell proliferation were indirectly assessed using the crystal violet (CV) assay, which is based on the staining of DNA and proteins in adherent cells [41,42,64]. MC3T3-E1 cells were seeded at a density of 5 × 10^3^ cells per well in 96-well cell culture plates. After 24 h of culture in DMEM, the culture medium was replaced with CM obtained from RAW 264.7 macrophage cultures, at concentrations of 100%, 50% and 25% (*v*/*v*), prepared by dilution with complete DMEM as described in Section 4.4, and the cells were incubated for an additional 72 h. The use of multiple concentrations of CM aimed to determine whether the biological effects of secretomes from different macrophage states are concentration-sensitive, as it has been shown that the concentration of CM derived from macrophages differentially affects osteoprogenitor proliferation and osteogenic differentiation [46,47]. Four repetitions were measured for each CM condition. Three corresponding DMEM control wells were averaged for normalization of each repetition. Following incubation, the CM and control medium was removed, and the adherent cells were fixed with methanol and stained with 1% CV solution (Sigma-Aldrich, St. Louis, MO, USA) for 10 min at room temperature. Excess dye was removed, stained cells were washed, and the bound dye was solubilized in 33% (*v*/*v*) acetic acid (Sigma-Aldrich, St. Louis, MO, USA). Absorbance was measured at 570 nm using a microplate reader. The absorbance of bound crystal violet, which reflects adherent cell biomass and cell number, was used as an indirect indicator of cell proliferation. Relative cell proliferation was expressed as a percentage of the control and calculated using the following formula: Relative cell proliferation (% of control) = (mean absorbance of CM-treated cells/mean absorbance of control cells) × 100.

### 4.8. In Vitro Osteogenic Differentiation of MC3T3-E1 Cells

MC3T3-E1 cells were seeded on glass coverslips at a density of 1 × 10^3^ cells per well in 24-well culture plates and cultured in DMEM for 24 h. The medium was then replaced with CMs collected from differently treated RAW 264.7 macrophage cultures, at concentrations of 100% and 25% (*v*/*v*), as explained in Section 4.4. Two concentrations of CM were used to compare undiluted CM with significantly diluted CM to determine whether osteogenic responses were altered with dilution, as has been previously shown that macrophage-CM concentration can modify osteoprogenitor responses [46,47]. Prior to use, each CM was supplemented with osteogenic supplements, namely 50 µM ascorbic acid 2-phosphate and 2 mM β-glycerophosphate (Sigma-Aldrich, St. Louis, MO, USA), based on previously described osteogenic culture protocols [65,66]. Control cells were cultured in either osteogenic medium (OM) or DMEM alone. The experiment was performed in quadruplicate, and the medium was replaced on day 3. The cultures were terminated on day 6, when the cells were fixed in 10% formalin for 15 min. The six-day endpoint was selected to assess early responses of MC3T3-E1 preosteoblasts to macrophage-derived CM, at a time when the CM-treated cultures had reached confluence or near-confluence under the applied seeding conditions. Following fixation, the cells were processed for cytochemical and immunocytochemical analyses.

### 4.9. Cytochemical Detection of Calcium-Containing Mineral Deposits

Calcium phosphate (CaP) deposition was indirectly assessed using *von Kossa* staining. Fixed cells were incubated with a 2% silver nitrate solution (Sigma-Aldrich, St. Louis, MO, USA) for 30–60 min under UV illumination. After washing, the cells were incubated with 5% sodium thiosulfate solution (Sigma-Aldrich, St. Louis, MO, USA) for 5 min. The samples were subsequently counterstained with Nuclear Fast Red solution (Sigma-Aldrich, St. Louis, MO, USA) for 1 min. Finally, the coverslips were mounted on microscopic slides and examined by light microscopy.

A semiquantitative analysis of CaP deposition was performed using ImageJ free software version 1.54k (National Institutes of Health, Bethesda, MD, USA). Four images were analysed for each group and concentration, acquired under 20× objective magnification. Images were converted to 8-bit grayscale, and areas corresponding to CaP deposition were identified by image thresholding. The percentage of the image area positive for mineral deposition was measured and expressed as CaP deposition (% area).

### 4.10. Immunocytochemical Detection of Osteogenic Markers

Primary antibodies against osteocalcin (anti-OCN, ab64261; Abcam, Cambridge, UK) and osteopontin (anti-OPN, ab8448; Abcam, Cambridge, UK) were used for immunocytochemical staining. Signal detection was performed using an HRP/DAB immunohistochemical (IHC) detection kit (ab64261; Abcam, Cambridge, UK), according to the manufacturer’s instructions.

Before staining, formalin-fixed cells were permeabilized with 0.25% Triton X-100 (Sigma-Aldrich, St. Louis, MO, USA) for 10 min. Endogenous peroxidase activity was blocked using the hydrogen peroxide blocking solution for 10 min, followed by incubation with the protein-blocking solution for 5 min at room temperature to reduce nonspecific staining. Separate cell samples were then incubated with primary antibodies against osteocalcin or osteopontin (each diluted 1:500) for 60 min at 37 °C. This was followed by incubations with a biotinylated secondary antibody and streptavidin-peroxidase complex each for 10 min at room temperature.

The immunoreactive signal was visualized using 3,3′-diaminobenzidine (DAB) chromogen (20 µL DAB in 1 mL substrate) for 10 min. Finally, the cells were counterstained with Mayer’s hematoxylin for 1 min and mounted using VectaMount Permanent Mounting Medium (Vector Laboratories, Peterborough, UK). Negative controls were processed identically, except that the primary antibody was omitted.

For quantification of OPN and OCN immunoexpression plugin IHC profiler in the ImageJ software was used. For each group and concentration, four images acquired under 20× objective magnification were analysed. ICC Optical Density (OD) score was calculated, using the following equation [67]: ICC OD score = (% high positive × 4) + (% positive × 3) + (% low positive × 2) + (% negative × 1).

### 4.11. Light Microscopy and Image Analysis

Cell morphology was monitored at different experimental time points using an inverted phase-contrast microscope (Axio Observer.Z1, Carl Zeiss, Oberkochen, Germany). Cytochemically and immunocytochemically stained samples were examined using a Leica DMLS light microscope (Leica Microsystems, Wetzlar, Germany) equipped with an AxioCam 105 color digital camera (Carl Zeiss, Oberkochen, Germany). Images were acquired using ZEN 2 software (blue edition; Carl Zeiss, Oberkochen, Germany).

### 4.12. Statistical Analysis

The original one-way analyses were performed using SPSS software, version 18.0 (SPSS Inc., Chicago, IL, USA). Data are presented as mean ± standard deviation (SD) or mean ± relative standard deviation (RSD), as indicated in each legend. RSD was calculated as (SD/mean) × 100% and describes dispersion relative to the group mean. The relative-to-control percentage plots retain the RSD designation, whereas the field-based image scores are reported with SD. Differences among groups were assessed using one-way analysis of variance (ANOVA). When ANOVA indicated a significant overall difference, Dunnett’s post hoc test was used to compare each treatment group with the untreated control group, whereas Tukey’s Honest Significant Difference (HSD) test was used for pairwise comparisons among the treatment groups. A value of *p* < 0.05 was considered statistically significant (* *p* < 0.05, ** *p* < 0.01, *** *p* < 0.001). For the MC3T3-E1 CV assay, four normalized repetitions per condition were analyzed as paired blocks in an exploratory two-factor repeated-measures ANOVA of macrophage condition (four levels), CM concentration (three levels), and their interaction. Greenhouse–Geisser-corrected *p* values were used for these effects, and pairwise comparisons were adjusted with the Holm method. DMEM was the normalization reference and was not included as a factor level. The *p* values describe only variability among the measured repetitions.

## 5. Conclusions

Our study indicates that macrophage-derived conditioned media exert a regulatory effect on osteogenesis in preosteoblastic MC3T3-E1 cells. The concentration of CM also modified the proliferation of preosteoblastic cells in a manner that depended on the state of the macrophages. At a concentration of 100% CM, both M(LPS)-CMs were associated with higher preosteoblasts proliferation than M(GC)-CM and Untreated-CM. However, dilution produced different effects: 25% M(LPS100)-CM significantly reduced proliferation compared to DMEM and the more concentrated M(LPS100)-CM, while diluted M(LPS1)-CM and M(GC)-CM supported greater proliferation than their 100% CM counterparts. However, CM obtained from macrophages activated by 1 ng/mL of LPS showed limited ability to produce prominent *von Kossa* staining or OCN immunoreactivity. In contrast, the 100 ng/mL M(LPS) condition showed more prominent *von Kossa* staining but weak OCN immunoreactivity. M(GC)-CM was associated with pronounced osteogenic differentiation, as evidenced by more prominent *von Kossa* staining and OCN immunoreactivity, and osteoblast-like morphology, together with the lowest mean proliferation value among the 100% CM concentrations. CM derived from untreated macrophages also supported osteogenic differentiation, consistent with an early osteogenesis-associated response to macrophage-derived factors. Among the tested conditions, M(GC)-CM showed the most pronounced overall osteogenic-associated pattern. These findings describe early responses assessed on day six, when cultures treated with CM were confluent or nearly confluent, but they do not confirm terminal osteoblast maturation or continuous matrix mineralization. No uniform dilution-dependent pattern was evident between 100% and 25% CM among different macrophage conditions. Different effects of CM from variously treated and untreated macrophages suggest that macrophage-derived signals differentially affect the balance between preosteoblast proliferation, differentiation, and ECM production under the tested conditions. This balance between proliferation and differentiation is crucial for developing effective strategies to enhance bone regeneration. It is also essential to precisely determine both the concentrations of the stimuli applied to macrophages and the concentration of CM subsequently applied to target cells, particularly when establishing pro-inflammatory macrophage activation conditions. Finally, our study supports the role of macrophages as key modulators in bone tissue engineering and highlights their potential in developing immunomodulatory strategies in the field of regenerative medicine. Future studies should explore the molecular pathways involved in these modulations, as well as evaluate the applicability of these findings in different in vivo wound healing and regeneration models.

## Figures and Tables

**Figure 1:**
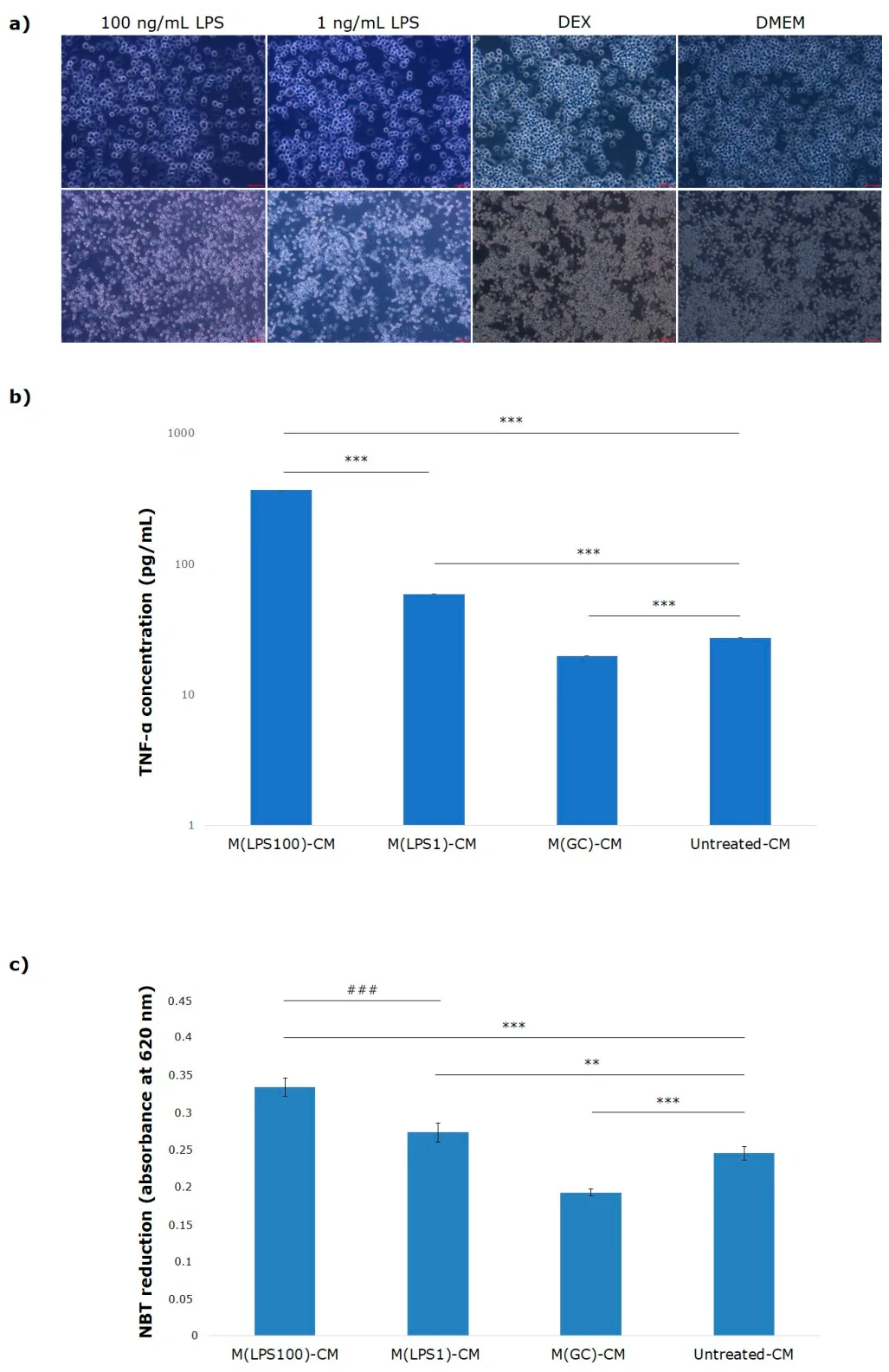
Macrophage characterisation: (**a**) Morphology of RAW 264.7 macrophages after the treatment with 100 ng/mL or 1 ng/mL LPS, dexamethasone (DEX) or left untreated (DMEM alone as control). Phase-contrast microscopy, objective magnification ×20, scale bar = 50 µm (the first row) and objective magnification ×10, scale bar = 100 µm (the second row). (**b**) TNF-α concentrations in conditioned media (CM) derived from differently treated RAW 264.7 macrophages: 100 ng/mL LPS (M(LPS100)-CM group), 1 ng/mL LPS (M(LPS1)-CM group), dexamethasone (M(GC)-CM group), or maintained in DMEM alone (Untreated-CM group, control), as determined by ELISA. Values are expressed as pg/mL. *** *p* < 0.001, (**c**) NBT reduction by differently treated RAW 264.7 macrophages under the same treatment conditions as described in (**b**). Bars represent the mean ± RSD. ** *p* < 0.01, *** *p* < 0.001 vs. control (Untreated-CM), ^###^ *p* < 0.001 M(LPS100)-CM vs. M(LPS1)-CM.

**Figure 2:**
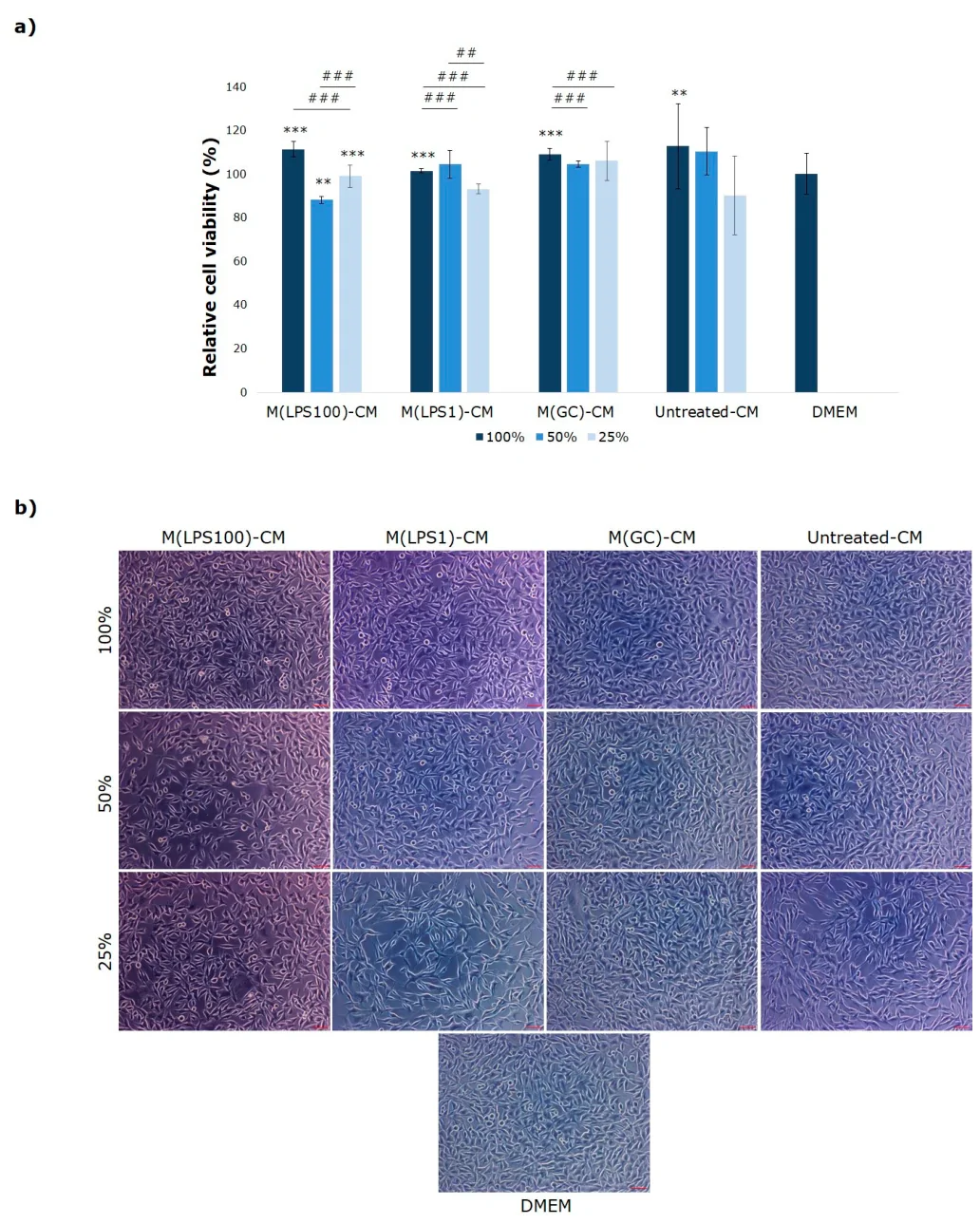
The L929 assay: (**a**) L929 cell viability cultured in 100%, 50% and 25% conditioned media (CM) from RAW 264.7 macrophages treated with: 100 ng/mL LPS (M(LPS100)-CM group), 1 ng/mL LPS (M(LPS1)-CM group), dexamethasone (M(GC)-CM group), or maintained without treatment (Untreated-CM group), as assessed by the MTT assay. Results are expressed as percentages relative to the DMEM control, set at 100%. Bars represent the mean ± RSD. ** *p* < 0.01 vs. DMEM, *** *p* < 0.001; ^##^ *p* < 0.01, ^###^ *p* < 0.001 between different concentrations inside the same group; ^††^ *p* < 0.01 50% M(LPS100)-CM vs. 50% Untreated-CM, 25% M(LPS100)-CM vs. 25% Untreated-CM; ^§§^ *p* < 0.01 50% M(LPS100)-CM vs. 50% M(LPS1)-CM, ^§§§^ *p* < 0.001 25% M(LPS100)-CM vs. 25% M(LPS1)-CM (^††^ *p*, ^§§^ *p* and ^§§§^ *p* data not presented in Figure 2a). (**b**) Phase-contrast micrographs of L929 cells after cultured in 100%, 50% and 25% conditioned media (CM) derived from RAW 264.7 macrophages treated with: 100 ng/mL LPS (M(LPS100)-CM group), 1 ng/mL LPS (M(LPS1)-CM group), dexamethasone (M(GC)-CM group), or maintained without treatment (Untreated-CM group). Cells cultured in DMEM alone served as the control. Phase-contrast microscopy, objective magnification ×20, scale bar = 50 µm.

**Figure 3:**
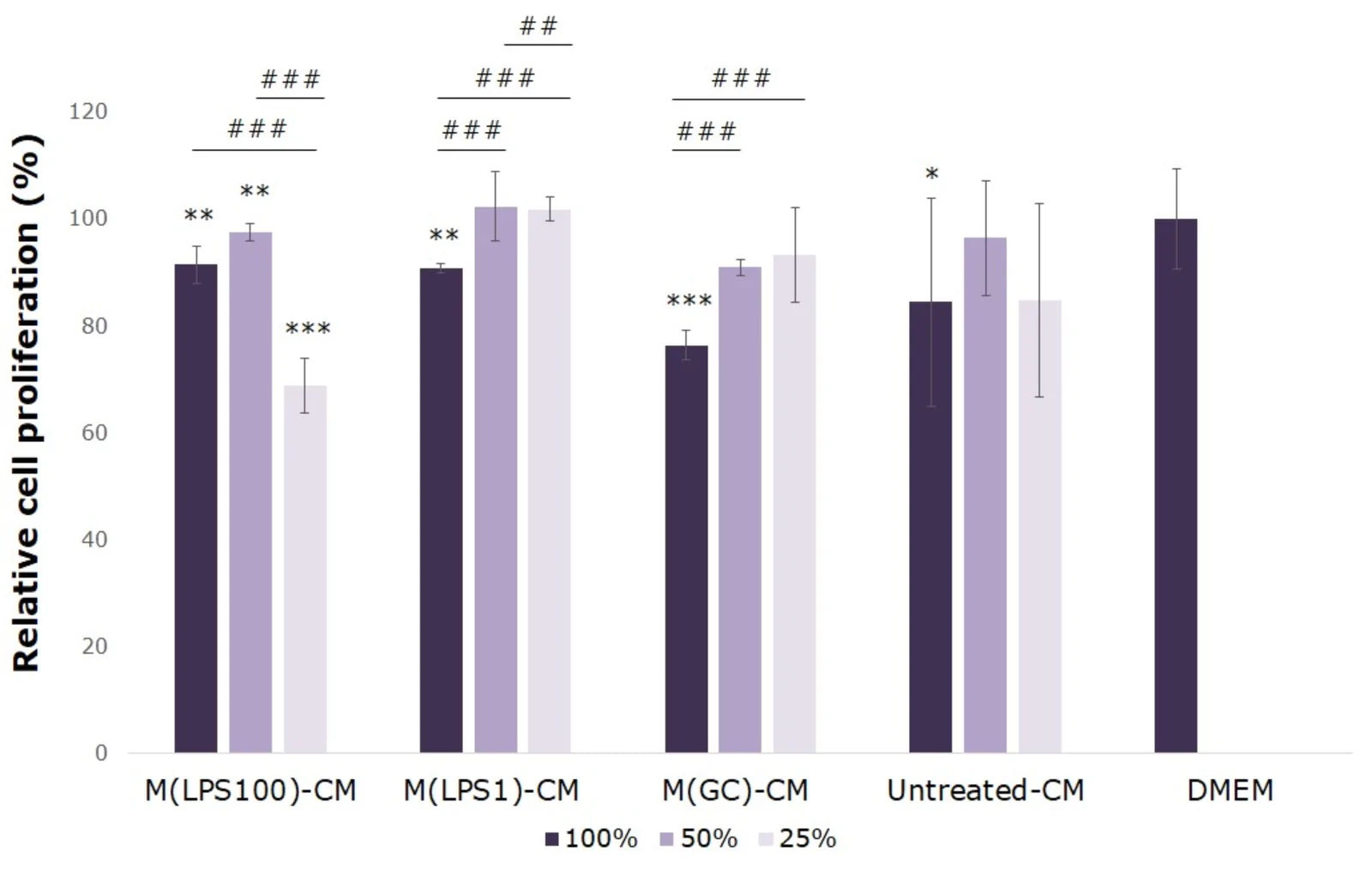
Relative CV signal as an indirect estimate of relative cell number or adherent MC3T3-E1 cell biomass after incubation in 100%, 50% and 25% conditioned media (CM) from RAW 264.7 macrophages treated with: 100 ng/mL of LPS (M(LPS100)-CM group), 1 ng/mL of LPS (M(LPS1)-CM group), dexamethasone (M(GC)-CM group), or maintained without treatment (Untreated-CM group). Results are expressed as percentages relative to the DMEM control, set at 100%. Bars represent the mean ± RSD. * *p* < 0.05, ** *p* < 0.01, *** *p* < 0.001 vs. DMEM; ^##^ *p* < 0.01, ^###^ *p* < 0.001 between different concentrations inside the same group; ^††^ *p* < 0.01 50% M(LPS100)-CM vs. 50% Untreated-CM, 25% M(LPS100)-CM vs. 25% Untreated-CM; ^§§^ *p* < 0.01 50% M(LPS100)-CM vs. 50% M(LPS1)-CM, ^§§§^ *p* < 0.001 25% M(LPS100)-CM vs. 25% M(LPS1)-CM (^††^ *p*, ^§§^ *p* and ^§§§^ *p* data not presented in Figure 3). Four repetitions were measured per CM condition.

**Figure 4:**
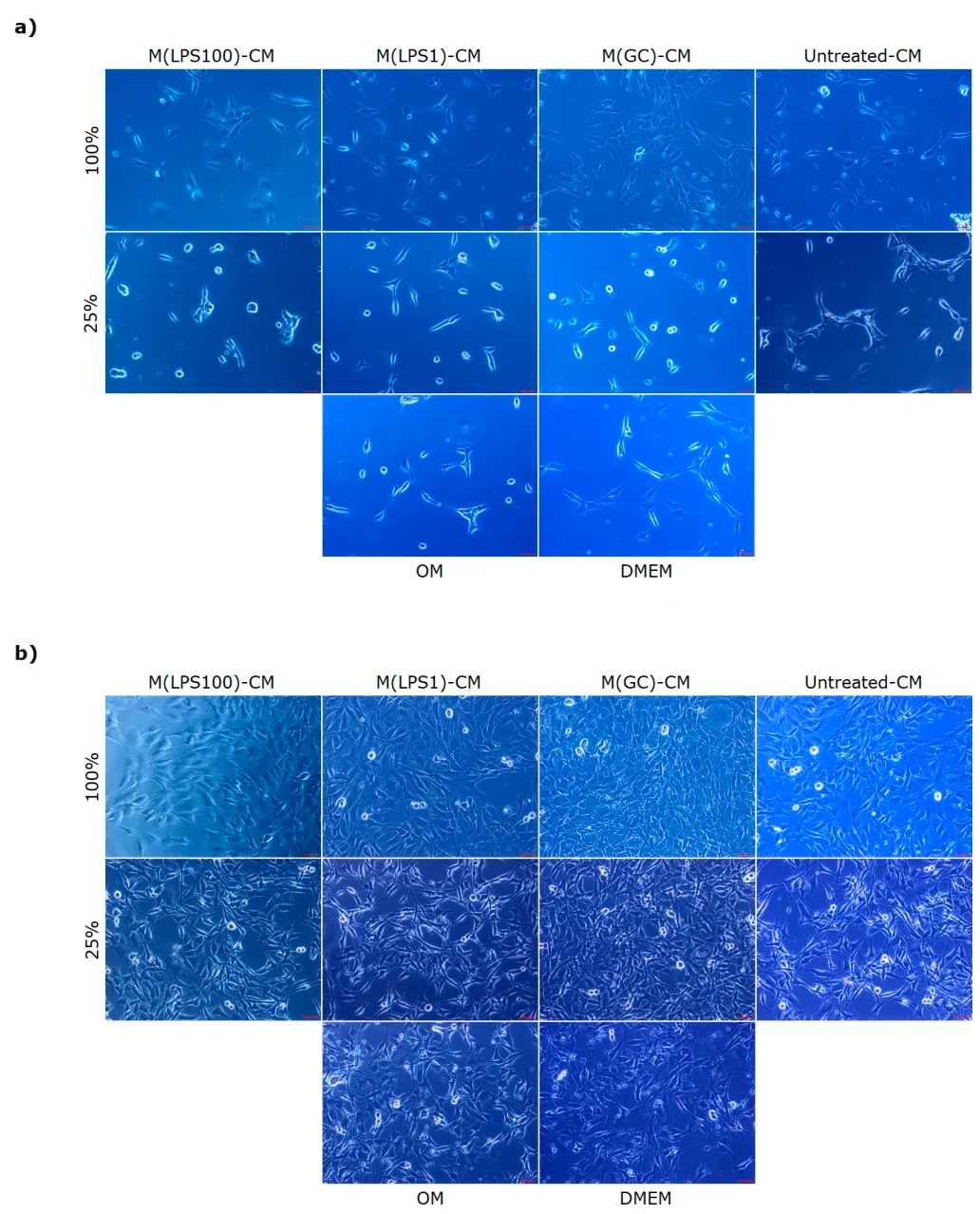
Temporal morphology of MC3T3-E1 cells: (**a**) Day 3 and (**b**) Day 6 of osteogenic differentiation in 100% and 25% conditioned media (CM) derived from RAW 264.7 macrophages treated with: 100 ng/mL LPS (M(LPS100)-CM group), 1 ng/mL LPS (M(LPS1)-CM group), dexamethasone (M(GC)-CM group), maintained without treatment (Untreated-CM group), osteogenic medium (OM) or DMEM alone. Phase-contrast microscopy, objective magnification ×20, scale bar = 50 µm.

**Figure 5:**
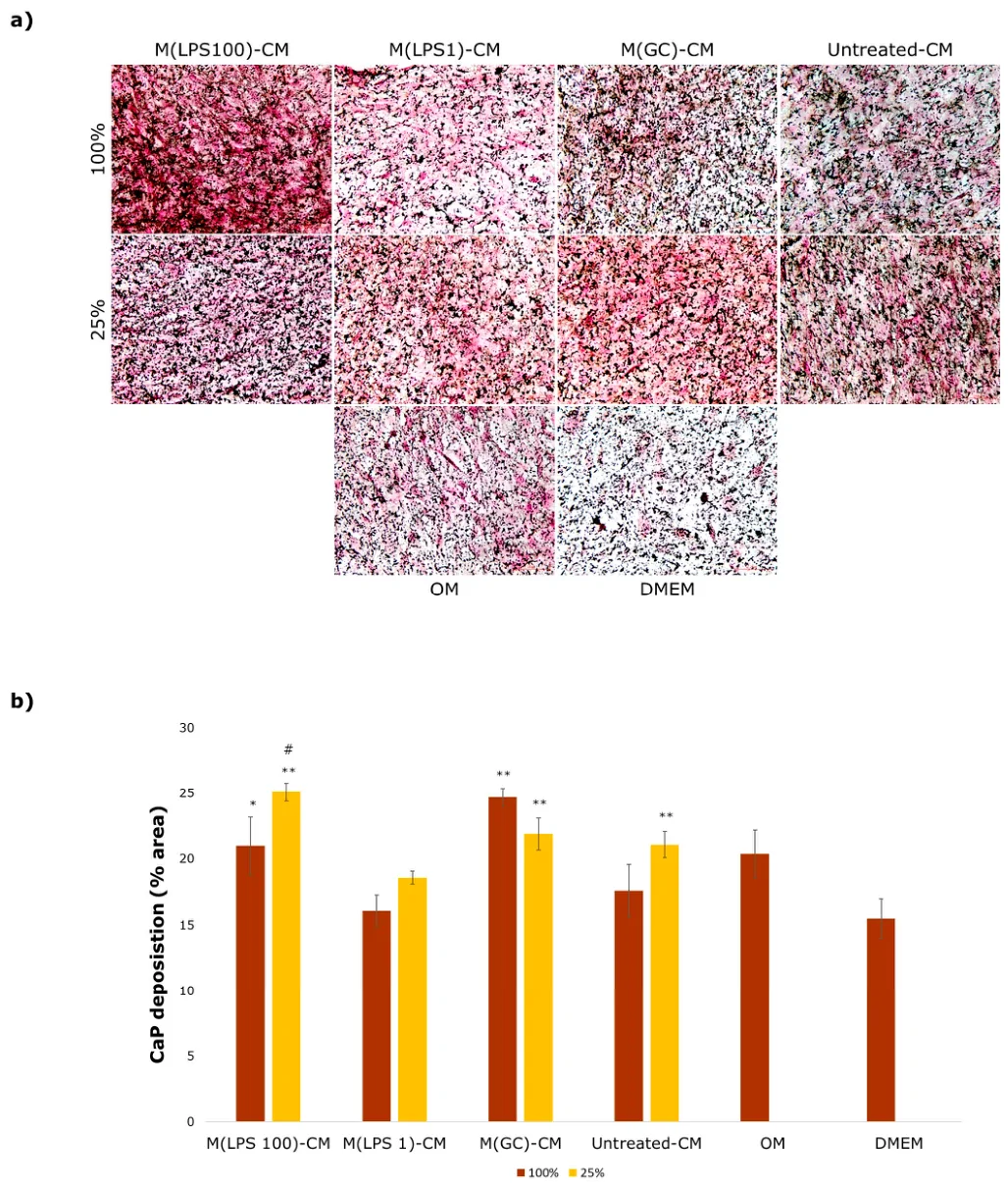
Calcium phosphate (CaP) deposition after 6 days of MC3T3-E1 cell osteogenic differentiation: (**a**) Detection of CaP deposits in 100% and 25% conditioned media (CM) derived from RAW 264.7 macrophages treated with: 100 ng/mL LPS (M(LPS100)-CM group), 1 ng/mL LPS (M(LPS1)-CM group), dexamethasone (M(GC)-CM group), maintained without treatment (Untreated-CM group), osteogenic medium (OM) or DMEM alone. *Von Kossa* staining, objective magnification ×20, scale bar = 50 µm. (**b**) Semiquantitative analysis of mineral deposition expressed as CaP deposition (% area) under the same differentiation conditions as described in (**a**). Bars represent the mean ± SD. * *p* < 0.05, ** *p* < 0.01 vs. DMEM; ^#^ *p* < 0.05 vs. OM; ^†^ *p* < 0.05 25% M(LPS100)-CM vs. 25% M(GC)-CM, 25% M(LPS1)-CM vs. 25% M(GC)-CM and 100% M(GC)-CM vs. 100% Untreated-CM, ^††^ *p* < 0.01 25% M(LPS100)-CM vs. 25% M(GC)-CM, ^†††^ *p* < 0.001 100% M(LPS1)-CM vs. 100% M(GC)-CM (^†^ *p*, ^††^ *p* and ^†††^ *p* data not presented in Figure 5b). Four analyzed image fields were used per group and concentration; the CaP-positive area was normalized to the analyzed field area.

**Figure 6:**
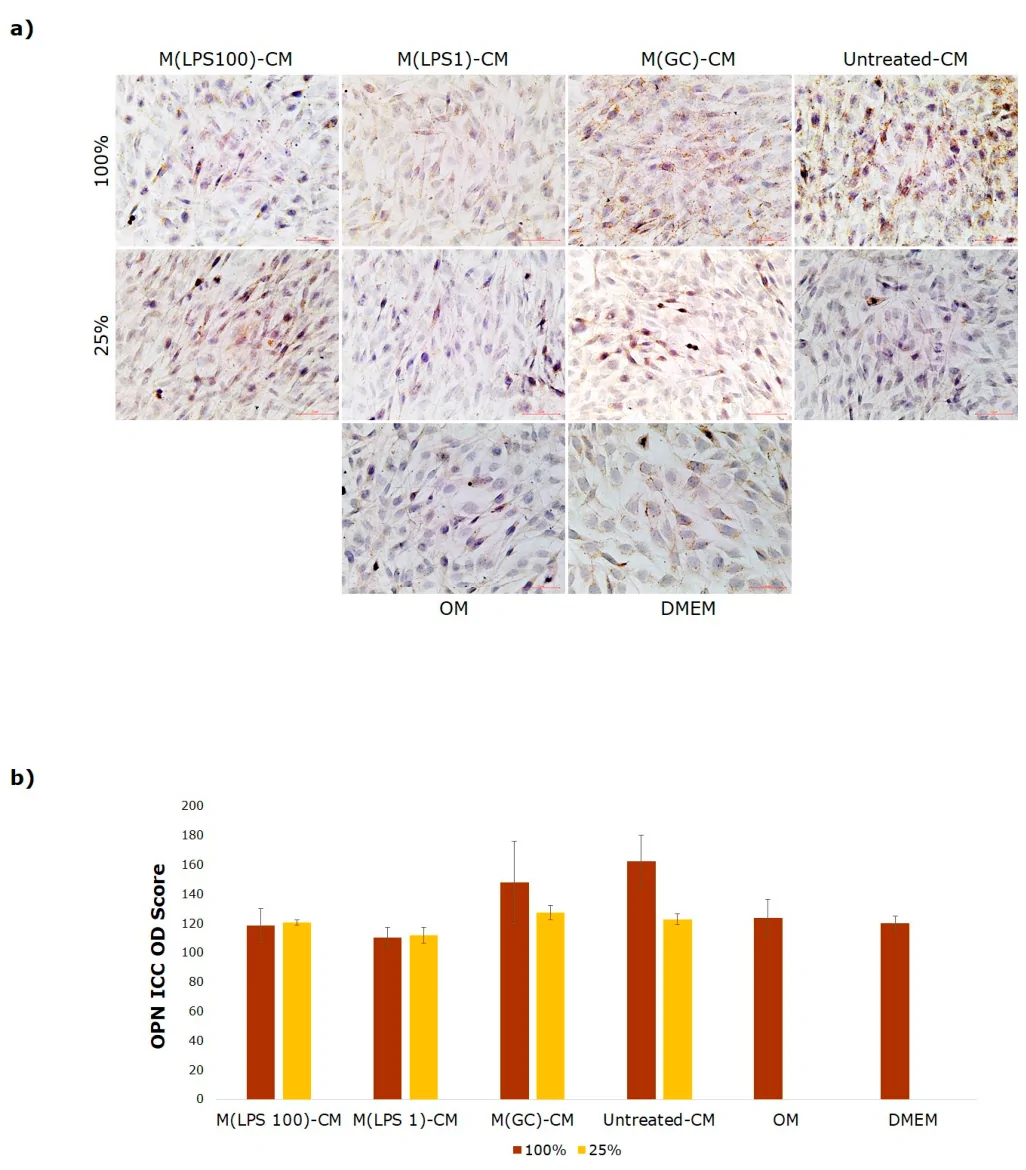
Osteopontin (OPN) immunocytochemical detection after 6 days of MC3T3-E1 cell osteogenic differentiation: (**a**) Immunocytochemical detection of OPN in 100% and 25% conditioned media (CM) derived from RAW 264.7 macrophages treated with: 100 ng/mL LPS (M(LPS100)-CM group), 1 ng/mL LPS (M(LPS1)-CM group), dexamethasone (M(GC)-CM group), maintained without treatment (Untreated-CM group), osteogenic medium (OM) or DMEM alone. Objective magnification ×20, scale bar = 50 µm. (**b**) OPN immunocytochemical optical density (OD) score under the same differentiation conditions as described in (**a**). Bars represent the mean ± SD. *p* < 0.05 25% M(LPS1)-CM vs. 25% M(GC)-CM and 100% M(LPS1)-CM vs. 100% Untreated-CM (*p* data not presented in Figure 6b). Four analyzed regions were used per group and concentration; staining categories were percentages of the analyzed field area.

**Figure 7:**
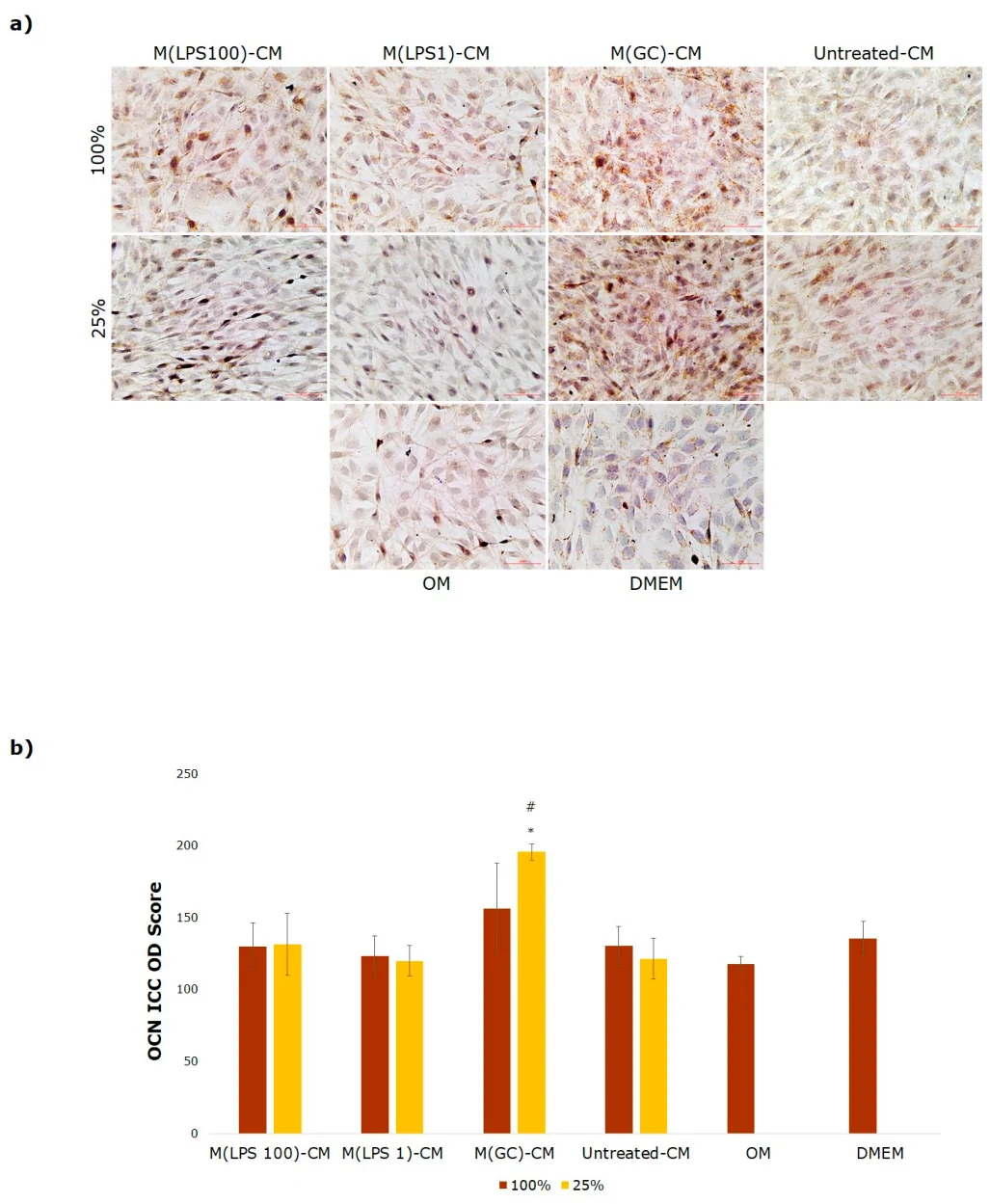
Osteocalcin (OCN) immunocytochemical detection after 6 days of MC3T3-E1 cell osteogenic differentiation: (**a**) Immunocytochemical detection of OCN in 100% and 25% conditioned media (CM) derived from RAW 264.7 macrophages treated with: 100 ng/mL LPS (M(LPS100)-CM group), 1 ng/mL LPS (M(LPS1)-CM group), dexamethasone (M(GC)-CM group), maintained without treatment (Untreated-CM group), osteogenic medium (OM) or DMEM alone. Objective magnification ×20, scale bar = 50 µm. (**b**) OCN immunocytochemical optical density (OD) score under the same differentiation conditions as described in (**a**). Bars represent the mean ± SD. * *p* < 0.05 vs. DMEM; ^#^ *p* < 0.05 vs. OM; ^†^ *p* < 0.05 25% M(LPS100)-CM vs. 25% M(GC)-CM; ^††^ *p* < 0.01 25% M(LPS1)-CM vs. 25% M(GC)-CM, ^†††^ *p* < 0.001 25% M(LPS1)-CM vs. 25% M(GC)-CM (^†^ *p*, ^††^ *p* and ^†††^ *p* data not presented in Figure 7b). Four analyzed regions were used per group and concentration; staining categories were percentages of the analyzed field area.

## Data Availability

All data are contained within the article.

## Abbreviations

The following abbreviations are used in this manuscript:

ALP	Alkaline Phosphatase
BMSCs	Bone Marrow Mesenchymal Stem Cells
BMP2	Bone Morphogenetic Protein 2
CaP	Calcium Phosphate
CM	Conditioned media
CPC	CaP Cement
DAB	3,3′-Diaminobenzidine
DEX	Dexamethasone
DMEM	Dulbecco’s Modified Eagle Medium
DMSO	Dimethyl sulphoxide
ECM	Extracellular matrix
FBS	Fetal Bovine Serum
ICC	Immunocytochemical
IL-1β	Interleukin-1 beta
LPS	Lipopolysaccharide
MSCs	Mesenchymal Stem Cells
MTT	3-(4,5-Dimethylthiazol-2-Yl)-2,5-Diphenyltetrazolium Bromide
NBT	Nitroblue Tetrazolium
OCN	Osteocalcin
OD	Optical Density
OM	Osteogenic medium
OPN	Osteopontin
OS	Osteogenic Medium
PMA	Phorbol 12-Myristate 13-Acetate
ROS	Reactive oxygen species
SD	Standard Deviation
TNF-α	Tumor Necrosis Factor-alpha
